# Advances in the roles of glycyrrhizic acid in cancer therapy

**DOI:** 10.3389/fphar.2023.1265172

**Published:** 2023-08-15

**Authors:** Yuqian Zhang, Zixuan Sheng, Jing Xiao, Yang Li, Jie Huang, Jinjing Jia, Xiansi Zeng, Li Li

**Affiliations:** ^1^ Research Center of Neuroscience, Jiaxing University Medical College, Jiaxing, China; ^2^ Department of Physiology, Jiaxing University Medical College, Jiaxing, China; ^3^ Department of Biochemistry and Molecular Biology, Jiaxing University Medical College, Jiaxing, China

**Keywords:** cancer, glycyrrhizic acid, anti-cancer, side effects, mechanisms

## Abstract

Since the first 70 years of reporting cancer chemotherapy, malignant tumors have been the second most common cause of death in children and adults. Currently, the commonly used anti-cancer methods include surgery, chemotherapy, radiotherapy, and immunotherapy. Although these treatment methods could alleviate cancer, they lead to different forms of side effects and have no particularly significant effect on prolonging the patients’ life span. Glycyrrhizic acid (GL), a native Chinese herbal extract, has a wide range of pharmacological effects, such as anti-cancer, anti-inflammatory, antioxidant, and immune regulation. In this review, the anti-cancer effects and mechanisms of GL are summarized in various cancers. The inhibition of GL on chemotherapy-induced side effects, including hepatotoxicity, nephrotoxicity, genotoxicity, neurotoxicity and pulmonary toxicity, is highlighted. Therefore, GL may be a promising and ideal drug for cancer therapy.

## 1 Introduction

Cancer has afflicted the multicellular organism for more than 200 million years, and the ancestors of modern humans were plagued by cancer more than a million years ago (Hausman, 2019). As the second most natural cause of death in children and adults (Gilbertson, 2011), each type of cancer has its particular age and form of occurrence, occurring in different proportions and in distinct genders. Until now, people have expended gigantic efforts to understand the origin of cancer cells, the formation of cancer tissue, and the mechanisms of their spread and recurrence, but this disease remains a mystery. A growing body of research suggests that the change in the incidence rate of cancer may be caused by more subtle alterations in the cell hierarchy (Lim et al., 2009; Molyneux et al., 2010). The formation of cancer probably involves three pathways: 1) spreading from the origin of growth to adjacent cells or tissues; 2) spreading from lymph nodes to local lymph nodes; 3) spreading to various tissues and organs throughout the body through blood circulation (Wong and Hynes, 2006).

Although the medical field has made some progress in cancer treatment after more than half a century of research, cancer is still deemed as a human tragedy. There are many treatment methods for cancer based on its type and progression stage. The traditional treatment methods for cancer include surgery, chemotherapy and radiotherapy. With the development of the times and technology, new cancer treatment methods, such as target-specific therapies and immunotherapy, are increasingly applied in clinical practice (Liu et al., 2021). However, these treatment methods all have certain limitations. Surgical treatment is highly traumatic and it cannot completely eliminate cancer cells. The effect of radiotherapy will be not ideal when tumors are not sensitive to radiation and tumors are of systemic metastasis. Among them, chemotherapy for malignant tumors has the strongest cancer killing effect (Safarzadeh et al., 2014; Jain et al., 2016). However, its toxic effects on healthy cells, multiple drug resistance (MDR) after permanent treatment and low bioavailability have enormously limited its clinical application (Rastogi et al., 2014). After these treatments, the survival time of cancer patients has been prolonged, but a great proportion of patients still experience cancer recurrence and cannot achieve permanently survival (Yin et al., 2021). Therefore, seeking novel methods for cancer treatment remains a crucial aspect of medical research.

## 2 Glycyrrhizic acid

### 2.1 The structure of GL

**FIGURE 1:**
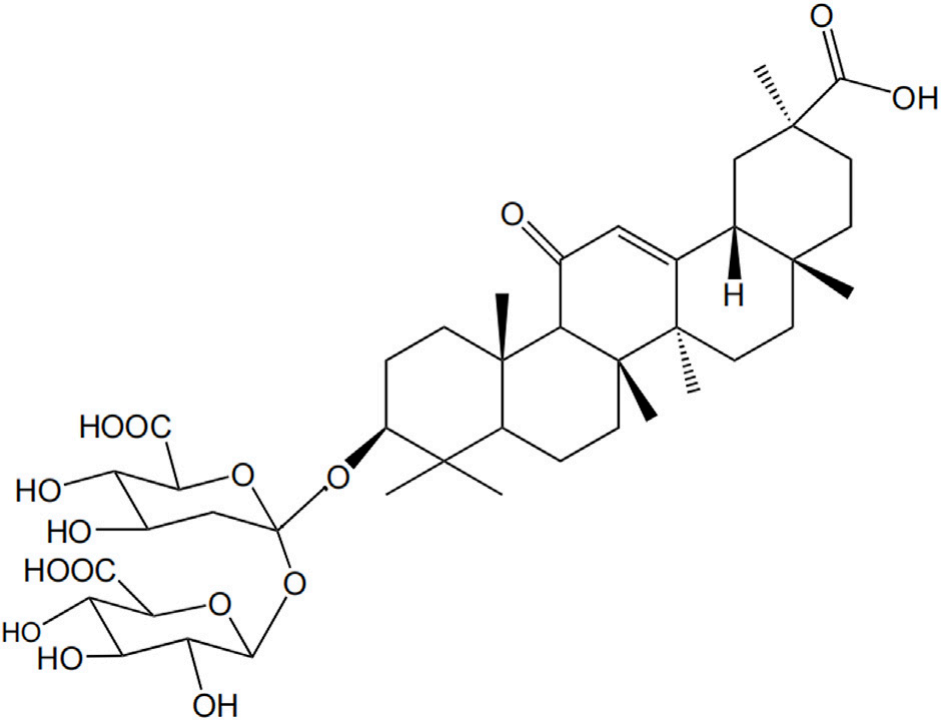
Structural formula of GL.

Glycyrrhizic acid (GL), a triterpenoid saponin, is the main active component and sweet component of the extract from the root of *Glycyrrhiza uralensis Fisch*. GL is structurally composed of two molecules of glucuronic acid and one molecule of glycyrrhetinic acid (GA) (Figure 1). GL is metabolized to GA under the action of gut bacteria. In addition, GL is metabolized in the intestine or be transformed via enzymolysis to GA-3-O-mono-β-d-glucuronide (GAMG), a distal glucuronic acid hydrolysate of GL with higher bioavailability and stronger physiological functions (Zuo et al., 2023).

### 2.2 The functions of GL

Accumulating studies have demonstrated that GL shows multiple pharmacological activities, such as anti-inflammatory (Liu et al., 2018; Maione et al., 2019), anti-tumor (Zhang et al., 2015; Chang et al., 2019; Tsai et al., 2020), anti-viral (Cinatl et al., 2003; Fu et al., 2016), antioxidant (Xu et al., 2018), immunoregulation (Bernela et al., 2016; Han et al., 2017) and liver protection (Huo et al., 2020) *etc.* GL mainly inhibits the expression of nuclear factor kappa-B (NF-κB) pathway, thereupon then restraining the encoding of important genes such as inflammatory cytokines, anti-apoptotic factors and cyclooxygenase-2 (COX2) (Li S. et al., 2014), achieving anti-inflammatory and anti-tumor effects. Thus, GL is commonly used in treating various cancers in clinical practice (Stecanella et al., 2021; Zuo et al., 2022), as well as in alleviating liver, lung, and kidney damage caused by cancer chemotherapy (Orazizadeh et al., 2014; Qu et al., 2017). Meanwhile, it also has a protective effect on brain tissue in cases of global cerebral ischemia, brain injury caused by cerebral hemorrhage, and focal ischemia (Xiong et al., 2016).

In addition, GL could facilitate the entry of other drugs into cells through interaction with cell membranes due to its strong hydrophilicity and lipophilicity (Selyutina and Polyakov, 2019). Studies have shown that even at high concentrations, GL still has pharmacological tolerance in rats and humans, and long-term administration has no significant toxic or side effects (van Rossum et al., 1999; Bi et al., 2023; Li et al., 2023). Therefore, it is often used as a blending agent for other drugs to diminish toxicity and enhance drug efficacy (Radwant and Aboul-Enein, 2002; Chen et al., 2009).

## 3 Roles of GL in cancer therapy

GL inhibits the occurrence and development of cancer by inducing apoptosis pathways in cancer cells. It shows a series of anti-cancer related pharmacological activities, such as broad-spectrum anti-cancer ability, anti-chemotherapy and radiotherapy induced tissue toxicity, absorption enhancement and anti-multiple drug resistance (MDR) mechanism (Wakamatsu et al., 2007; Ajazuddin et al., 2014). Multiple studies have shown that GL acts as an inhibitor of cell signal transduction molecules, angiogenesis inhibitors, tumor related cytokine inhibitors, efficiently and low-toxicity inhibiting the migration and invasion of several types of cancer cells (Kohlschutter et al., 2008; Smolarczyk et al., 2012). The following sections will specifically highlight the anti-cancer mechanism of GL and its inhibition on the side effects of cancer treatment.

### 3.1 Anti-cancer effects of GL

GL has been reported to have inhibitory effects on various cancers, such as leukemia (Chueh et al., 2012; Bruserud et al., 2015; Shustik et al., 2017), malignant glioma (Li S. et al., 2014), colon cancer (Yang et al., 2015; Jiang et al., 2016), lung cancer (Khan et al., 2013; Huang et al., 2014) *etc.* The application of GL in various cancers was summarized in Table 1.

**TABLE 1:** Summary of anti-cancer mechanisms of GL.

Cancer type	Models	Administration method and dosage of GL	The efficacy of GL	The anticancer mechanism of GL	References
Colon Cancer	Wistar rats	Orally, 15 mg/kg for 15 weeks	Anti-tumor; Anti-inflammation	Reducing the expression of Ki-67, proliferating cell nuclear antigen (PCNA), NF-kB, COX-2, iNOS, VEGF; enhancing the expression of p53, connexin-43, Bcl-2, survivin, and caspase-3	Khan et al. (2018)
	Balb/c mice	intraperitoneally, 50 or 100 mg/day for 20 days combined with bacteria overexpressing β-glucuronidase	Anti-tumor	Inhibiting tumor growth, increasing apoptosis rate	Afkhami-Poostchi et al. (2020)
Gastric Cancer	KATO III cells	3 mg/mL for 72 h	Anti-tumor	Inhibiting the growth of KATO III cells; Inducing apoptosis	Hibasami et al. (2005)
	MGC-803 cells	1 mg/mL for 24 and 48 h	Anti-tumor	Down-regulating the expression of G1 phase related proteins; Inhibiting phosphorylation of the PI3K/AKT pathway; Downregulating the expression of Bcl-1, survivin and p65, and Up-regulating the expression of Bax and the cleavage of PARP	Wang et al. (2020)
	AGS cells	200 µM for 4 h	Antibacterial infection	Inhibiting the expression of HMGB1; Restoring autolysosomal degradation function	Khan et al. (2023)
Leukemia	WEHI-3 cells	200, 250, 300, 350 and 400 μM GL and for 24 and 48 h	Anti-tumor	Increasing the levels of ROS and the activity of caspase-3; decreasing the mitochondrial membrane potential (ΔΨm); leading to G0/G1 phase arrest, DNA damage and breakage	Chueh et al. (2012)
	BALB/c mice suffering TF-1 cells	100 mg/kg body weight (peritumorally once every other day)	Anti-tumor	Reducing the activity of AKT, mTOR, and STAT3 in tumors; Attenuating expression of cyclin D1, survivin and increasing cleaved caspase-3, cleaved PARP expression	He et al. (2015)
	K562 cells/EL-4 lymphoma-bearing C57BL/6J mice	1.0–4.0 mM for 48 h/50 or 500 mg/kg/day injected i.p. for 4 days	Anti-tumor	Enhancing the levels of apoptosis in K562 cells; Decreasing the tumor burden in mice	Hostetler et al. (2017)
Glioblastoma	human glioblastoma U251 cells	0, 1, 2, 4 mM for 1, 2 or 4 days	Anti-tumor; Anti-inflammation	Inhibiting the proliferation; Down-regulating the expression of p65	Li et al. (2014)
Lung Cancer	A549 cells/A549 cell xenograft in nude mice	1, 2 mM for 3 days/50 mg/kg every 2 days for 8 weeks	Anti-tumor	Inhibiting the growth of A549 cells and inducting apoptosis; Inhibiting expression of TxAS	Huang et al. (2014)
	HCC827 cell xenograft in nude mice	100 mg/kg for 2 weeks	Anti-tumor	Inhibiting the growth of HCC827 cells; Targeting JAK/STAT/HMGB1 signaling	Wu et al. (2018)
Hepatoma	HepG2 and PLC/PRF/5	0.5, 1, and 2 mM for 48 h	Anti-tumor	Inhibiting tumor growth through inducing differentiation and repressing stemness; Enhancing the anti-tumor effects of sorafenib	Cai et al. (2019)
	SK-Hep1 and Hep3B cells; SK-Hep1/luc2 tumor-bearing mice	40 or 80 μM for 48 h; 50 mg/kg/day by intraperitoneal injection for 7 days	Anti-tumor	Inhibiting tumor cell growth, cell invasion, and expression of AKT, ERK, EGFR phosphorylation, anti-apoptotic and metastatic proteins; triggering caspase-8/9-mediated apoptosis	Tsai et al. (2020)
Melanoma	Murine B16F10 melanoma; human A375 melanoma cells	5, 10, 20, 30, 40, 50, 100 ug/mL for 24 h	Anti-tumor	Down-regulating the expression of Treg specific marker, pSTAT3, COX2, PGE2, Bcl2; Up-regulating the expression of Bax and inducing cell apoptosis; Inhibiting of the pSTAT3-mediated Immunosuppressive function of Tregs and MDSCs	Juin et al. (2020)
	Mice inoculated with B16F10 melanoma cells	10 mg/kg for 1, 3, 5 and 7 days	Anti-tumor	Inhibiting the pulmonary metastases of B16 melanoma	Kobayashi et al. (2002)

#### 3.1.1 Anti-colon cancer

Colon cancer, a disease with high incidence, easy migration and difficult cure, constantly develops drug resistance during chemotherapy, increasing the difficulty of treatment (Liang et al., 2020). Khan et al. (2018) found that GL administration inhibited the 1,2-dimethylhydrazine-induced colon tumorigenesis in Wistar rats through reducing the expression of Ki-67, proliferating cell nuclear antigen (PCNA), NF-kB, cyclooxygenase-2(COX-2), inducible nitric oxide synthase (iNOS), and vascular endothelial growth factor (VEGF) and enhancing the expression of p53, connexin-43, B-cell lymphoma-2 (Bcl-2), survivin, and caspase-3, suggesting the chemopreventive potential of GL against colon cancer. Simultaneously, upregulation of the expression of tumor suppressor protein p53 activates casepase-3, thereby inducing tumor cell apoptosis (Yi et al., 2016). In addition, GL reduced drug resistance by combining with first-line anti-cancer drugs. Some theories suggest that GL may enhance the absorption of paclitaxel-loaded GL micelles in the jejunum and colon by inhibiting p-glycoprotein (Yang et al., 2015). Interestingly, Afkhami-Poostchi et al. (2020) applied bacteria-directed enzyme prodrug therapy to convert GL to glycyrrhetinic acid and found that combined treatment of bacteria overexpressing β-glucuronidase and GL more greatly inhibited tumor growth when compared with sole GL treatment.

#### 3.1.2 Anti-gastric cancer

Gastric cancer is the fifth leading cause of cancer-related deaths worldwide, with approximately half of cases occurring in developing countries (Ang and Fock, 2014; Karimi et al., 2014; den Hoed and Kuipers, 2016). GL could inhibit the growth of stomach cancer KATO III cells and induce the fragmentation of DNA to oligonucleosomal-sized fragments, suggesting that GL induced apoptosis (Hibasami et al., 2005). Wang et al. (2020) reported that GL induced apoptosis MGC-803 cells of inhibiting phosphorylation of the PI3K/AKT pathway, downregulating the expression of Bcl-1, survivin and p65, and upregulating the expression of Bax and the cleavage of poly (ADP-ribose) polymerase (PARP). Cell cycle arrest could cause inhibition of cell proliferation. A study has shown that GL inhibited the proliferation of MGC-803 cells, a kind of gastric cancer cells, by inducing G1/s phase arrest because GL treatment downregulated the levels of several G1 phase-related proteins (cyclin D1, D2, D3, E1, and E2) (Wang et al., 2020). In a recent study, Khan et al. (2023) reported that GL treatment inhibited *helicobacter pylori* infection in AGS cells (gastric cancer cells) via inhibiting high mobility group box1 (HMGB1) and inducing autolysosomal degradation function.

#### 3.1.3 Anti-leukemia

Leukemia is one of the causes of cancer-related deaths in humans, and is an invasive malignant tumor, that is, produced by the rapid growth of abnormal white blood cells (Lee et al., 2007). GL treatment increased the levels of reactive oxygen species (ROS) and the activity of caspase-3 and decreased the mitochondrial membrane potential (ΔΨm) in WEHI-3 cells, as well as led to G0/G1 phase arrest, DNA damage and breakage in a dose-dependent manner (Chueh et al., 2012). GL could inhibit TF-1 cells proliferation *in vitro* and reduce the volume of TF-1 tumor via inhibiting the activation of AKT/mTOR/signal transducer and activator of transcription 3 (STAT3) signaling pathway, attenuating the expression of cyclin D1 and survivin and increasing the cleavage of caspase-3 and PARP (He et al., 2015). In addition, GL reversed multidrug resistance in human leukemia cell line CEM/ADR 5000 (Zhou and Wink, 2018). Based on these anti-leukemia capabilities, researches on the rationality of GL combination therapy are gradually being carried out. A study has shown that co-treatment with GL and imatinib (a first-line drug) not only enhanced the levels of apoptosis greatly in K562 cells (chronic myeloid leukemia), but also decreased the tumor burden significantly in EL-4 lymphoma-bearing C57BL/6J mice (Hostetler et al., 2017).

#### 3.1.4 Anti-glioblastoma

Gliomas are the most common brain tumor of the central nervous system, approximately accounting for 35%–50% of adult intracranial tumors. It is worth noting that malignant gliomas account for about 60% of gliomas (Penas-Prado et al., 2012). Research has shown that GL inhibited the proliferation of human glioblastoma U251 cells in a time- and dose-dependent manners via down-regulating the expression of p65 (Li S. et al., 2014). NF-κB is a key transcription factor involved in the pathological processes of various human diseases, controlling multiple genes involved in the development of diffuse gliomas and promoting the growth of high-grade gliomas (Kanzawa et al., 2003; Wang et al., 2004). GL inhibited NF-κB pathway via downregulating the expression of p65 protein, encoding of important genes such as anti-inflammatory cytokines, COX-2 and iNOS (Li S. et al., 2014). Dipotassium glycyrrhizinate, a dipotassium salt of GL, also showed an anti-proliferative effect via inducing apoptosis and an anti-migratory effect in U251 and U138MG cells through upregulating the levels of miR-4443 and miR-3620, which are responsible for the post-transcriptional inhibition of NF-κB (Bonafe et al., 2022a).

#### 3.1.5 Anti-lung cancer

Lung cancer is the most common malignant tumor worldwide and the main cause of death for cancer patients. Huang et al. (2014) found that GL inhibited the growth of A549 cells by induction of apoptosis, but had no effects on NCI-H23 cells, another lung adenocarcinoma cell line. They further reported that GL suppressed the expression and activity of thromboxane synthase (TxAS) in A549 cells and clarified the anti-tumor effect of GL in lung adenocarcinoma cells is dependent on inhibition of TxAS (Huang et al., 2014). Importantly, treatment with 50 mg/kg GL for 8 weeks significantly inhibited the growth of xenograft of lung adenocarcinoma cells *in vivo* (Huang et al., 2014). Deng et al. (2017) reported that the effect of GL treatment alone is comparable to combination of GL and cisplatin in a mouse lung adenocarcinoma model, suggesting the clinical application of GL may decrease the dosage of cisplatin, thereby reducing the side effects of chemotherapy. In HCC827 cells, a non-small cell lung cancer cell line, GL inhibited the migration and invasion of cancer cells via targeting JAK/STAT/HMGB1 signaling (Wu et al., 2018).

#### 3.1.6 Anti-hepatoma

Liver cancer is a common malignant tumor with extremely high mortality rate, ranking third among global cancer mortality rates (Sung et al., 2021). Through the research on the prevalence of liver cancer, it is found that the incidence rate of liver cancer in most countries around the world is still rising year by year. Hepatocellular carcinoma (HCC) has poor differentiation ability and proliferate indefinitely. The dedifferentiation of HCC contributes to malignant progression, characterized by significant morphological changes and loss of liver function (Sun et al., 2011). A recent study has shown that GL administration led to a decrease in stem cell pluripotency and induced the differentiation in HCC *in vitro* and *in vivo* by targeting c-Jun N-terminal kinase 1 (JNK1) (Cai et al., 2019); Importantly, blockage of JNK1 mitigate the degree of malignancy of HCC (Cai et al., 2019). In addition, GL combination effectively enhanced the anti-tumor effects of sorafenib, an inhibitor of multi kinases, in HCC treatment (Cai et al., 2019). Tsai et al. (2020) reported that GL not only inhibited dramatically the tumor cell growth and invasion, as well as the phosphorylation of extracellular-signal-regulated kinase (ERK), Akt (Ser473), epidermal growth factor receptor (EGFR) and the anti-apoptotic and metastatic proteins, but also triggered markedly caspase-8/9-mediated apoptosis in HCC *in vitro* and *in vivo*. The combination of GL could reverse the resistance of cisplatin in hepatocellular carcinoma cells via inhibiting of MDR-associated proteins (Wakamatsu et al., 2007). Covalent conjugation of GL with polyethyleneimine increased significantly the gene transfection efficiency and superior selectivity for HepG2 cells, suggesting the potential clinical application *in vivo* (Cao et al., 2019). Several studies reported that co-delivery of GL enhanced the therapeutic efficacy of doxorubicin for hepatocellular carcinoma *in vitro* and *in vivo* (Wang et al., 2019; Yang et al., 2019), suggesting the significant clinical application value.

#### 3.1.7 Anti-melanoma

Melanoma is a highly malignant tumor with pigmented cell, melanocyte, accounting for less than 5% of all skin cancers but 80% of skin cancer-related deaths (Bertolotto, 2013). The incidence of malignant melanoma has been steadily increasing globally over the past few decades (Godar, 2011). The immunosuppressive tumor microenvironment (TME) has been identified as a major barrier to evoke an anti-tumor response in melanoma. Moreover, immunosuppressive TME is directly connected with the high activation of T-regulatory cells (Tregs) and myeloid-derived suppressor cells (MDSCs) function. GL downregulated the expression of the anti-apoptotic factor Bcl 2, upregulated the expression of the proinflammatory factor Bax and enhanced the activity of caspase-9 and caspase-3, indicating that GL inhibited the proliferation of melanoma cells by inducing apoptosis (Juin et al., 2020). Meanwhile, GL incubation effectively reduced the expression of Treg specific markers (Forkhead Box P3, glucocorticoid-induced TNFR-related protein and cytotoxic T lymphocyte antigen 4), phospho-STAT3, COX-2 and prostaglandin E2 in melanoma cells, finally limiting the progression of melanoma (Juin et al., 2020). Furthermore, studies showed that GL inhibited the metastasis of melanoma cells by regulating T helper type 2 (Th2) cell resistance, interfering with further the dissemination of melanoma (Kobayashi et al., 2002; Su et al., 2017). Dipotassium of GL inhibited the metastases of melanoma Cells into brain (Bonafe et al., 2022b).

**FIGURE 2:**
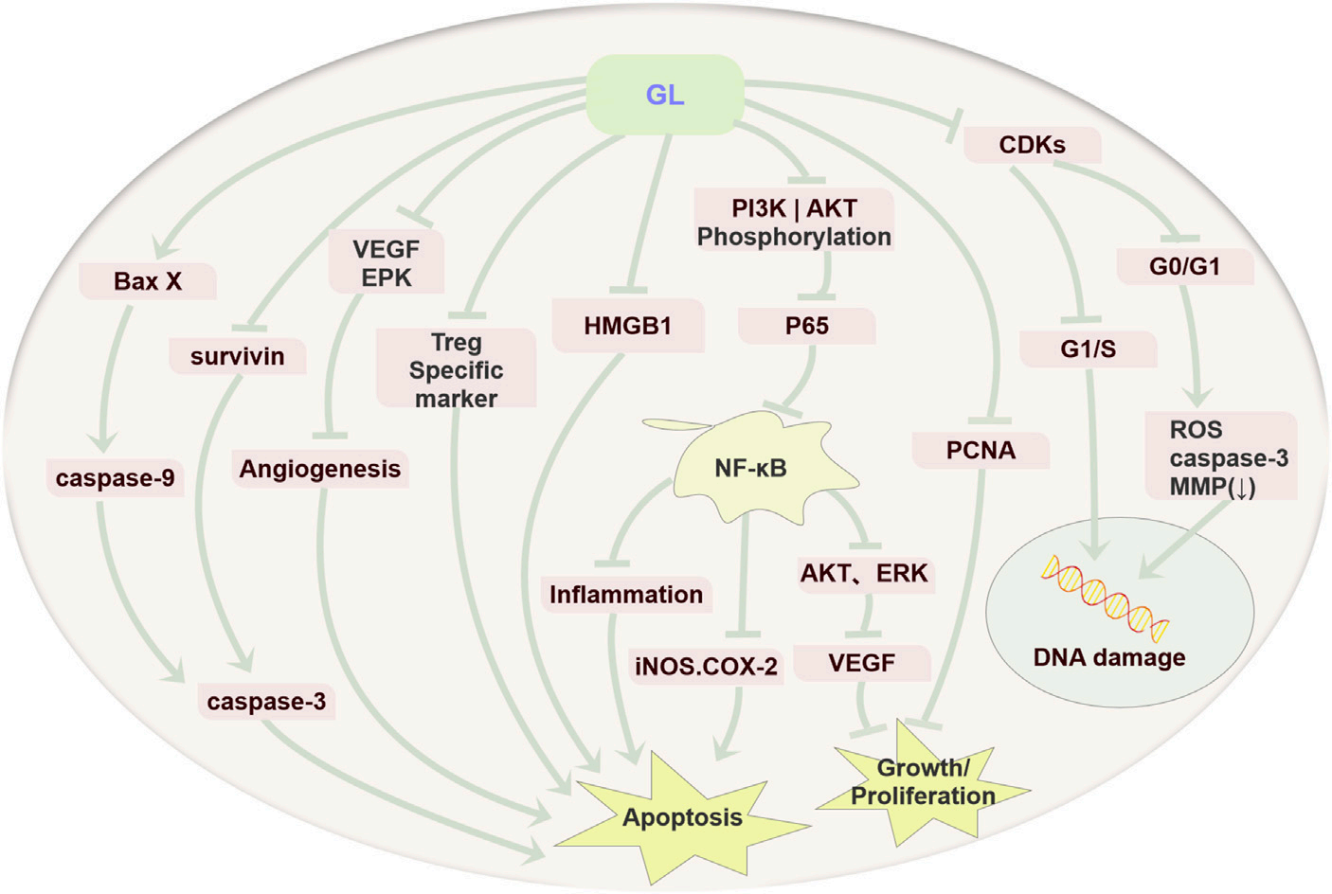
Anti-cancer mechanism diagram of GL. GL mainly plays an important role in various cancers by inhibiting the proliferation of cancer cells, inducing cell apoptosis through its anti-inflammatory, antioxidant, and immune regulatory effects, and inducing cell cycle arrest.

To summarize, GL exerts the anti-tumor activity via inhibiting cell proliferation, inducing apoptosis and resulting in cell cycle arrest in various tumors (Figure 2).

### 3.2 Inhibiting the side effects of cancer treatment

**FIGURE 3:**
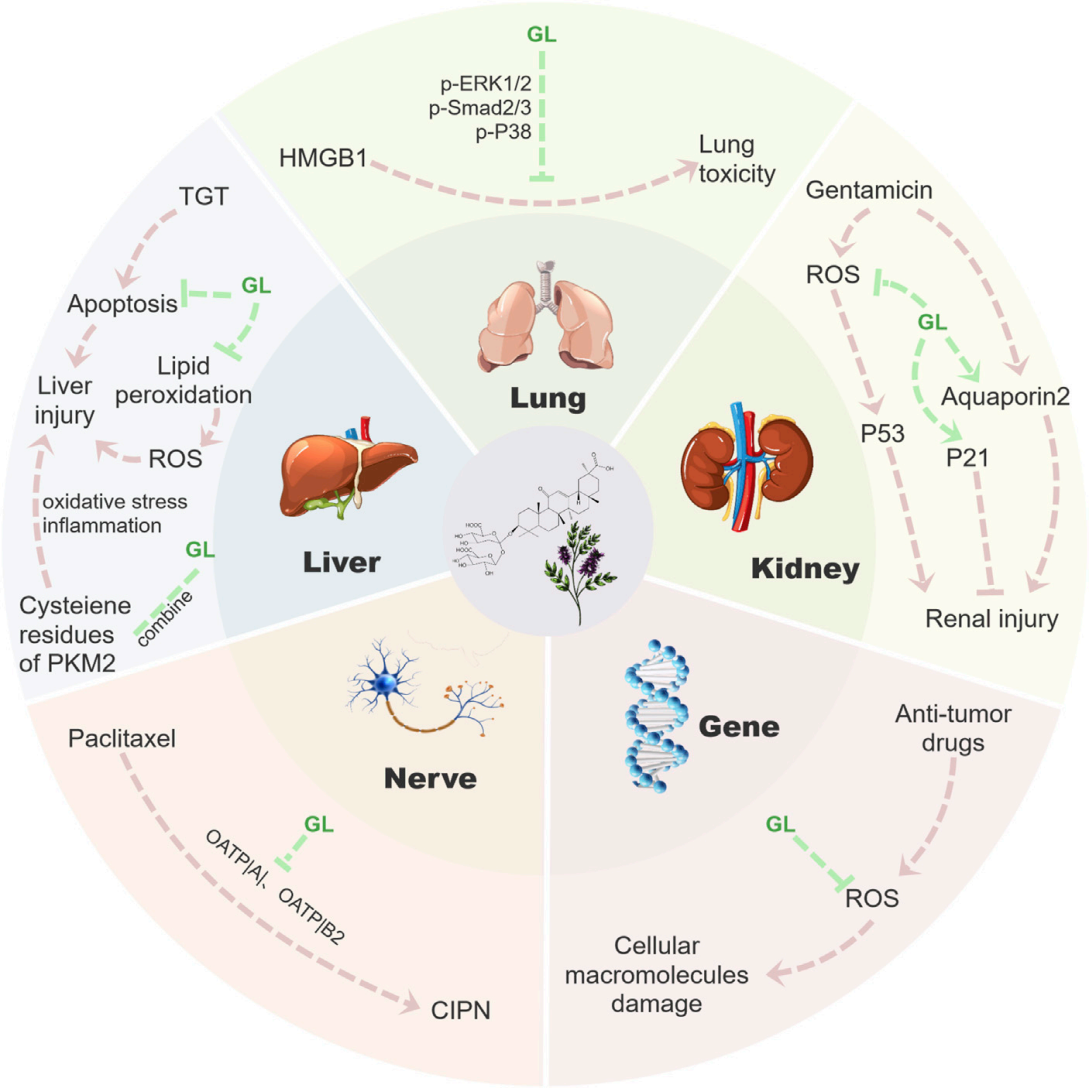
Mechanism diagram of GL reducing toxic side effects. GL could inhibit a series of side effects such as nephrotoxicity, hepatotoxicity, genotoxicity, neurotoxicity and lung toxicity in cancer treatment by inhibiting the release of inflammatory factors, inhibiting apoptosis and clearing ROS. Fundamentally speaking, GL mainly reduces a series of tissue damage in the process of tumor treatment through its anti-inflammatory and antioxidant properties, thus effectively reducing the side effects of tumor treatment.

Most anti-cancer drugs could cause inevitable damage to normal cells during treatment. Therefore, the protection of normal tissues and organs becomes the primary goal of improving the life quality of patients. An increasing number of studies have shown that GL has a strong therapeutic effect on liver, lung and kidney injury caused by chemotherapy or radiotherapy. In addition to the above anti-cancer capabilities, GL could significantly reduce side effects occurring during chemotherapy, especially hepatotoxicity (Li J. Y. et al., 2014). In addition, GL also has better therapeutic effects on nephrotoxicity (Ju et al., 2017), genotoxicity (Arjumand and Sultana, 2011), neurotoxicity (Klein et al., 2023), pulmonary toxicity (Zhu et al., 2021) and other events occurring during cancer treatment. In the following section, we will review the inhibition and potential mechanisms of GL on these side effects (Figure 3).

#### 3.2.1 Inhibition of hepatotoxicity

Liver is the most important organ for the metabolism of chemical drugs. After metabolized in the liver, a large number of metabolites accumulates in the liver and lead to liver damage and hepatotoxicity. The action mechanism is by inducing the liver lipid peroxidation, resulting in imbalance in redox status, at the same time high levels of ROS-mediated oxidative stress reaction cause liver tissue cell apoptosis and inflammation damage, eventually lead to hepatotoxicity (Waseem et al., 2015). GL administration for 3 h before high-dose methotrexate administration significantly increased hepatic enzyme levels (Mano et al., 2023), suggesting that optimal administration of GL could avoid pharmacokinetic interactions with methotrexate and exerts a hepatoprotective effects. Hepatotoxicity is a common toxic side effect of many chemotherapeutic drugs. Kishimoto et al. (2021) reported that 39 of 118 patients with acute leukemia (33%) developed grade 3–4 hepatotoxicity after combined administration of intravenous monoammonium glycyrrhizinate and methotrexate. As one of the hepatoprotective compounds, GL is commonly used in the treatment of acute and chronic liver injury, viral hepatitis, hepatic steatosis, liver fibrosis, liver cancer and other diseases. Moreover, GL has various pharmacological effects, such as anti-inflammatory, neuroprotection, antiviral, anti-tumor, antioxidant, and hepatoprotective activities (Li J. Y. et al., 2014). It could reduce the content of ROS by reducing lipid peroxidation (Kiso et al., 1984) and increasing the activity of superoxide dismutase (SOD), glutathione peroxidase (GPx) and catalase (Kao et al., 2009). Furthermore, GL inhibits tripterygium-glycoside-tablet-induced acute liver injury by regulating pyruvate kinase M2 and reducing oxidative, inflammation stress and apoptosis (Wang et al., 2022). In addition, GL could significantly reduce the steatosis and necrosis of hepatocytes, inhibit interstitial inflammation and liver fibrosis, and promote cell regeneration (Li J. Y. et al., 2014).

#### 3.2.2 Inhibition of nephrotoxicity

Chemotherapy is one of the main cancer treatments. In cancer treatment, traditional chemotherapeutic drugs are the first-line drugs for the treatment of several malignancies, but they also cause kidney toxicity. In addition to traditional cytotoxic drugs, molecular targeted drugs could also affect kidney function and cause nephrotoxicity, which limits the efficacy of treatment and affects the life quality and overall survival of patients (Santos et al., 2020). Studies have shown that the nephrotoxicity induced by some anti-cancer drugs may be closely related to the oxidative stress and inflammatory responses. GL has antioxidant and anti-inflammatory properties, which could effectively inhibit pro-inflammatory substances (NF-κB, TNF-α, IL-1β, IL-6, and HMGB 1), increase levels of nuclear factor erythroid 2-related factor 2 (Nrf 2) and heme oxygenase-1 (HO-1), and restore the activity of antioxidant enzyme and GSH/GSSG ratio (Wu et al., 2015), reduce lipid peroxidation (Arjumand and Sultana, 2011), suggesting that GL effectively inhibits renal inflammation and oxidative stress, and relieve chemical kidney injury. In gentamicin-induced renal injury, GL may inhibit gentamicin-induced ROS generation or scavenge ROS before it reaches the cellular target tissue, while GL also restores the expression of aquaporin 2, thus improving renal defects in rats with gentamicin-induced acute renal failure (Sohn et al., 2003). Moreover, GL could also ameliorate cisplatin-induced renal injury and inhibit nephrotoxicity through reducing ROS-mediated p53 activation and promoting p21 expression in HK-2 cells (Ju et al., 2017). The renal protective effects of GL may be related to upregulation of Nrf2 and downregulation of NF-κB in the kidney of BALB/c mice (Wu et al., 2015).

#### 3.2.3 Inhibition of genotoxicity

Most of the anti-tumor drugs have genotoxic effects that contribute to growth inhibition. Exposure to genotoxins causes an increased risk of carcinogenic and teratogenicity (Arjumand and Sultana, 2011). These genotoxic and oncogenic potential may lead to the formation of secondary cancers (Said Salem et al., 2017). Some studies have indicated that many drug-induced genotoxicity and chromosomal instability are closely related to the parameters of oxidative stress. ROS act directly on intracellular components, including lipids, proteins and DNA, and disrupt their structures. The chemoprotective agents could exert their antigenotoxic effects through one or variety of mechanisms, such as inhibiting the formation of reactive carcinogenic metabolites, inducing enzymes that detoxify carcinogens, scavenging ROS, inhibiting cell proliferation and regulating cell apoptosis (Arjumand and Sultana, 2011). GL has anti-inflammatory, anti-oxidant, anti-cancer, and immunomodulatory effects (Akamatsu et al., 1991; Kelloff et al., 1994; Wakamatsu et al., 2007) and is considered as a possible chemopreventive agent. Increasing genotoxic researches have demonstrated that GL is not only nonteratogenic and nonmutagenic, but also have anti-genotoxic property (Isbrucker and Burdock, 2006). Arjumand and Sultana (2011) reported that GL administration significantly reversed the genotoxicity induced by cisplatin, including a decrease in DNA fragmentation and increases in the content of glutathione and activities of the anti-oxidant enzymes (catalase, glutathione peroxidase, glutathione reductase, quinone reductase and glutathione-S-transferase).

#### 3.2.4 Inhibition of neurotoxicity

Cancer therapies could cause a wide range of neurologic adverse effects and may cause a significant increase in morbidity and mortality of cancer patients (Dietrich, 2020). Some chemotherapeutic drugs, such as taxanes (paclitaxel and docetaxel), platinum compounds (cisplatin, oxaliplatin and carboplatin), and vinca alkaloids (vinblastine and vincristine), have strong neurotoxicity and could easily induce a peripheral neuropathy (CIPN) (Klein and Lehmann, 2021). CIPN is associated with a length-dependent axonal sensory neuropathy in taxol-induced neuropathy (Staff et al., 2017). Paclitaxel easy access to and accumulate in the dorsal root ganglia, which will cause numbness and pain in hands and feet (Cavaletti et al., 2000). Paclitaxel induces disruption of axonal transport through microtubule stabilization, changes in mitochondrial morphology and function, and inflammatory responses, leading to axonal symmetry damage and loss of nerve fibers, and subsequent neurotoxicity (Niznansky et al., 2022). Increasing studies have suggested that GL could provide neuroprotection in nerve system due to its strong anti-inflammatory, anti-apoptosis, antioxidant and autophagy regulation properties (Kim et al., 2012; Yang et al., 2018; Gendy et al., 2023; Shan et al., 2023). A recent study has shown that GL alleviated paclitaxel-induced neurotoxicity *in vitro* and *in vivo* by inhibiting the neuronal uptake mediated by organic anion transport peptides (OATPs), which are the main neuronal transporters of paclitaxel (Klein et al., 2023).

#### 3.2.5 Inhibition of pulmonary toxicity

Some chemotherapeutic agents, such as bleomycin, could cause severe pulmonary fibrosis and produce pulmonary toxicity (van der Schoot et al., 2016). GL could alleviate benzo(a)pyrene exposure-induced lung injury in rats via ameliorating the detoxification and antioxidant function of lung (Qamar et al., 2012). HMGB1 is a cytokine-like protein found in the nucleus of all cells and has multiple functions in inflammation, infection, tissue damage, cell apoptosis, and immune response (Qu et al., 2019). Furthermore, HMGB1 could not only act as a proinflammatory factor to directly involve in tissue damage (Qin et al., 2006), but also induce lung fibrosis through NF-κB-mediated release of transforming growth factor beta1 (TGF-β1) (Wang et al., 2017). As an inhibitor of HMGB 1, GL treatment reduced the inflammation and fibrosis by inhibiting the mitogen-activated protein kinase (MAPK) inflammatory signaling and Smad3 fibrotic signaling pathway (Zhu et al., 2021), thereby alleviating the lung toxicity induced by bleomycin, which is clinically used to treated various tumors (Gederaas et al., 2023).

## 4 Conclusion and expectation

With the development of modern science and technology, the discovery of plant pharmacological components was promoted. GL, as the main active ingredient of the licorice extract, has a wide range of pharmacological activities. Notably, increasing literature have demonstrated that GL shows inhibitory effects on various cancers by inhibiting cell proliferation, inducing apoptosis and resulting in cell cycle arrest through multitudinous mechanisms. What’s more, GL could reduce effectively the side effects of cancer treatment via inhibition of chemotherapy-induced renal toxicity, liver toxicity, genotoxicity, neurotoxicity and pulmonary toxicity. Given the important roles of GL in cancer treatment, the application of GL alone in the cancer chemotherapy or combined use with other anti-tumor drugs will have very bright application prospects.

Besides above side effects induced in the chemotherapy of cancer, ototoxicity is another common side effect which limits the clinical use of chemotherapeutics (Basirat et al., 2023). Many chemotherapeutics could affect the inner ear or auditory nerve, leading to hearing loss. For example, Cisplatin is widely used as a chemotherapeutic drug with a high rate of ototoxicity (an average incidence of more than 60%) (Arwanda et al., 2023). Inflammation and oxidative stress may be closely related to the ototoxicity of Cisplatin (Ramkumar et al., 2021). However, the role of GL in combating ototoxicity induced by chemotherapy is largely unclear. Therefore, it is expected that GL will help solving the hearing loss caused by ototoxic drugs in the future. What’s more, the mechanisms for GL inhibiting various side effects are not presently clarified accurately. Thus, more studies should be performed to elucidate the protective mechanisms.

GL shows stronger actions in cancer treatment, but some factors may limit its clinical application. The human body has multiple reactions to GL and there are significant individual differences. However, the reasons for the differences are not yet clear, and the proportion of sensitive populations has not been determined. On the basis of different administration methods and drug concentrations, electrolyte imbalance, edema, elevated blood pressure and false aldosterone symptoms may occur (Gomez-Sanchez and Gomez-Sanchez, 1992; Johns, 2009; Celik et al., 2012). Studies have shown that the risk of toxicity after oral administration is much lower than intravenous or intraperitoneal administration, but we should still be particularly careful to avoid high doses or long-term ingestion of GL (Wang and Nixon, 2001). What’s more, people with diseases such as heart and kidney problems, hypertension may be more susceptible to the adverse effects of GL (Ruszymah et al., 1995). In addition, the content of GL in different varieties of liquorice varies greatly, and the yield depends on the source of plants, which also limits greatly its development and application. Han et al. (2021) recommended that regulating biosynthesis pathway of GL through environmental stimuli would provide a new idea for obtaining high-quality GL. Although side effects of GL have been reported, their incidence can be avoided with reasonable medication. The most important thing is that its potential for treating various diseases is worthy of recognition.
