# Corrigendum: Advances in the roles of glycyrrhizic acid in cancer therapy

**DOI:** 10.3389/fphar.2023.1345663

**Published:** 2023-12-14

**Authors:** Yuqian Zhang, Zixuan Sheng, Jing Xiao, Yang Li, Jie Huang, Jinjing Jia, Xiansi Zeng, Li Li

**Affiliations:** ^1^ Research Center of Neuroscience, Jiaxing University Medical College, Jiaxing, China; ^2^ Department of Physiology, Jiaxing University Medical College, Jiaxing, China; ^3^ Department of Biochemistry and Molecular Biology, Jiaxing University Medical College, Jiaxing, China

**Keywords:** cancer, glycyrrhizic acid, anti-cancer, side effects, mechanisms

In the published article, there was an error in **Affiliations 1 and 2**. Instead of “^1^Research Center of Neuroscience, Jiaxing, China; ^2^Department of Physiology, Jiaxing, China,” it should be “^1^Research Center of Neuroscience, Jiaxing University Medical College, Jiaxing, China; ^2^Department of Physiology, Jiaxing University Medical College, Jiaxing, China.”

The authors apologize for this error and state that this does not change the scientific conclusions of the article in any way. The original article has been updated.

